# Indoor Human Action Recognition Based on Dual Kinect V2 and Improved Ensemble Learning Method

**DOI:** 10.3390/s23218921

**Published:** 2023-11-02

**Authors:** Ruixiang Kan, Hongbing Qiu, Xin Liu, Peng Zhang, Yan Wang, Mengxiang Huang, Mei Wang

**Affiliations:** 1 School of Information and Communication, Guilin University of Electronic Technology, Guilin 541004, China; bbklasnic@glut.edu.cn; 2 Ministry of Education Key Laboratory of Cognitive Radio and Information Processing, Guilin University of Electronic Technology, Guilin 541004, China; 3 College of Information Science and Engineering, Guilin University of Technology, Guilin 541004, China; liuxin2017125@glut.edu.cn (X.L.); 1020210969@glut.edu.cn (M.H.); mwang@glut.edu.cn (M.W.); 4 State Grid Qianshan City Electric Power Supply Company, Qianshan 246300, China; 21021201011@mails.guet.edu.cn; 5 Northwest Survey and Planning Institute of the National Forestry and Grassland Administration, Xi’an 710048, China; wychong@glut.edu.cn

**Keywords:** human action recognition, Kinect V2, ensemble learning, fuzzy c-means algorithm, random forest, binocular system

## Abstract

Indoor human action recognition, essential across various applications, faces significant challenges such as orientation constraints and identification limitations, particularly in systems reliant on non-contact devices. Self-occlusions and non-line of sight (NLOS) situations are important representatives among them. To address these challenges, this paper presents a novel system utilizing dual Kinect V2, enhanced by an advanced Transmission Control Protocol (TCP) and sophisticated ensemble learning techniques, tailor-made to handle self-occlusions and NLOS situations. Our main works are as follows: (1) a data-adaptive adjustment mechanism, anchored on localization outcomes, to mitigate self-occlusion in dynamic orientations; (2) the adoption of sophisticated ensemble learning techniques, including a Chirp acoustic signal identification method, based on an optimized fuzzy c-means-AdaBoost algorithm, for improving positioning accuracy in NLOS contexts; and (3) an amalgamation of the Random Forest model and bat algorithm, providing innovative action identification strategies for intricate scenarios. We conduct extensive experiments, and our results show that the proposed system augments human action recognition precision by a substantial 30.25%, surpassing the benchmarks set by current state-of-the-art works.

## 1. Introduction

In the Internet of Things (IoT) era, more and more sensor networks are playing an undeniable role in various situations, such as agricultural greenhouses [1], warehouses [2], outdoor parking lots [3], outdoor hazardous environments [4], and indoor classrooms [5]. Beyond tangible economic advantages, these networks exert a profound influence on societal dynamics. Notably, several studies emphasize sensors integrated with Software Development Kits (SDKs), attracting significant global enthusiasm and attention.

Human Action Recognition (HAR) is usually considered as capturing and obtaining the action data via sensors, subsequently facilitating identification across diverse scenarios [6]. HAR can be an essential process, which can be adopted as core parts in human–computer interaction systems, smart city systems, IoT home management systems, and so on [1,2,3,4,5,6,7,8,9,10,11,12,13,14,15,16,17,18,19,20,21,22]. Within the realm of HAR-enabled systems, primary devices can be categorized into contact-type (wearable) and non-contact devices. Notably, several studies are centered around the former. In [8], Zheqi Yu et al. use inertial measurement unit (IMU) sensors and Universal Software Radio Peripheral (USRP) devices to complete multimodal data interaction and improve the accuracy of HAR. Jingcheng Chen et al. use various wearable devices to collect user data for HAR tasks [9]. Heilym Ramirez et al. integrate Alpha Pose and IMU devices for system architecture and data acquisition, emphasizing fall detection through skeleton joint data analysis [10]. Mohamed E. Issa et al. use various contact-type devices to collect human data, and later different methods are adopted in the medical sector [11]. However, judging by the scenarios mentioned above, contact-type devices are increasingly exposing their shortcomings. Some users believe that such devices that must be worn on time are neither comfortable nor convenient. Additionally, many of these devices demand robust battery support or alternative backup solutions [3,4,5,6,7,8,9,10]. There may be privacy issues and sensitive permission controversies, which greatly hinder the promotion of these IoT devices.

As the technological landscape evolves, there has been a growing emphasis on non-contact devices. Kinect Devices [12,13] (Microsoft Corp., Redmond, WA, USA), Leap Motion [5,14] (Leap Motion Inc, San Francisco, CA, USA), Halcon [3,15] (MVTec Software GmbH, Munich, Bavaria, Germany) et al. have emerged as frontrunners in this domain. Functioning akin to traditional depth cameras, these devices are adept at capturing RGB data, depth map information, and more. In addition, their capability to amass multimodal heterogeneous data tailored to specific application realms greatly streamlines research implementation and development globally. Lixiong Gong et al. use a Halcon camera and optical flow field method to track vehicles in outdoor environments [3]. Nicolas Octavio Medina Chilo et al. use Leap Motion devices in [4]. They are proven to be suitable for Exploration Ordnance Disposal (EOD) tasks. Jesús Galván-Ruiz et al. use Leap Motion to finish Spanish sign language action recognition [16]. The Kinect series offers a broader spectrum of utility. Notwithstanding the discernible differences in design, core parameters, power provisions, and other elements across the Kinect V1 (also known as Microsoft Kinect), Kinect V2, and Kinect V3 (also known as Azure Kinect), developers globally have been enthusiastic about integrating the camera and microphone arrays of these devices. Leveraging their unique SDKs, they have orchestrated multimodal or audio–visual interactive projects. Their strides in this arena have culminated in notable achievements. In [13], Min Li et al. use one Kinect V1 to collect infant sleep posture data for early cerebral palsy, and complete the detection, thus obtaining predictive references for specific diseases in medical scenarios. Fangfang Gao et al. use Kinect V2 to collect crop RGB images and point cloud data for an automated picking system [1]. Dong Wei et al. use Azure Kinect to collect joint data and RGB data for human–computer intention detection [17]. Although Kinect devices may not be as specialized as other depth cameras in some aspects, they are highly cost-effective and have shining points that continue to have profound influences.

The advent of deep learning has revolutionized the field of vision-based systems, particularly in action recognition. While some scenarios may have fixed orientations, existing systems now employ both traditional machine learning models and deep learning models to fulfill HAR tasks. These models include Convolutional Neural Network (CNN)-based methods [7,10,23], Recurrent Neural Network (RNN)-based methods [24], Graph Convolutional Neural Network (GCN)-based methods [25,26,27], Bidirectional Encoder Representation from Transformers (BERT)-based methods [28,29], Transformer-based methods [29], and Generic Adversarial Networks (GAN)-based methods [28,30]. For instance, Sagar Chhetri et al. have utilized optical flow field theory and CNN-based methods to detect falls in elderly individuals [23], achieving excellent results with reduced detection time. Andrea Apicella et al. have employed CNN-RNN and RGB images to detect falls in the elderly [24]. Ke Cheng et al. have proposed a novel shift-GCN network to address the inflexibility and uncontrollability of spatial and temporal graph receptive fields, reducing computational complexity while performing HAR tasks based on skeleton data [25]. As for the skeleton joint data, PYSKL [26] and DG-STGCN [27] et al. have been proposed and obtain excellent results. Moreover, the BERT and Transformer models, originally used in Natural Language Processing (NLP) projects, have been gradually applied to HAR tasks with fixed perspectives. BERT-GAN [28] and MotionBERT [29] have achieved significant results in HAR tasks, while GAN-based methods [28,30] have been proposed to address imbalanced multi-classification applications in different situations. It is undeniable that the methods mentioned above and datasets including NTU-60, NTU-120 and others [31,32,33,34,35,36,37,38,39,40,41], which contain various actions, have also played a crucial role in facilitating research. This is not only reflected in the application scenario for a single-person issue but also in the application scenario for multiple persons working simultaneously. The deep learning models proposed have greatly promoted the research. Their impact is evident to all of us. However, these related works mostly focus on research from a fixed perspective. Once the position of the receiver or depth camera is fixed, its position cannot be changed during the recognition process. This poses a challenge when users unintentionally choose an unexpected perspective to complete an action, as it is difficult to ensure that the aforementioned models can still perform as expected. At the same time, the huge computational burden is also an undeniable factor that constrains cross-platform development projects. Among them, the limitations of monocular systems are fundamental. Although a large number of sample sets and training may alleviate this drawback, it greatly limits their applications in flexible orientation scenarios.

**Figure 1:**
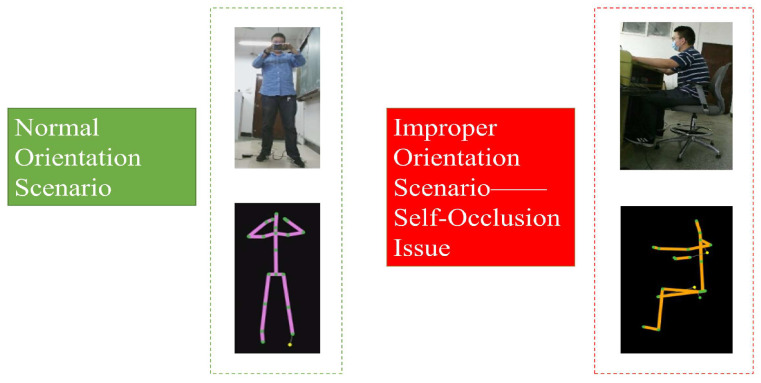
The Joint Connection in Different Orientations.

Nevertheless, monocular systems coupled with flexible orientation challenges have presented inherent limitations. Notably, the official joint connection mechanism from Kinect V2 is susceptible to self-occlusions, particularly when multiple parties interact. Such occlusions can significantly undermine the recognition process [5,20,22]. This phenomenon will make the connections among joints erratic and unpredictable, which will have an enormous negative impact on HAR tasks. Furthermore, improper orientation increases the likelihood of encountering incorrect or confusing skeleton joint connections. Consequently, these irregularities in skeleton joint connections can render identification methods within monocular systems ineffective. The descriptions are as follows (Figure 1):

In complex situations, additional measures and processes are required to handle other multimodal information in the system. A heightened emphasis on acoustic localization signals, as derived from Kinect and other IoT devices, is imperative [1,2,3,4,5,6,7,8,9,10,11,12,13,14,15,16,17,18,19,20,21,22,33,34]. If dynamic obstacles appear in the transmission path, NLOS scenarios may occur in an indoor localization system. It may result in scattering, diffraction, or irregular energy decay in the transmission path. This will make the signal transmission function and the processing methods originally designed for line-of-sight (LOS) scenarios no longer applicable. The localization aspect introduced in our dual Kinect V2 system is a key part of the compensation process for self-occlusion issues in complex situations. When confronted with NLOS acoustic signals, it becomes essential to adopt efficacious strategies to ensure heightened accuracy in indoor localization. Therefore, our core works are as follows:

(1) We introduce a novel dual Kinect V2 binocular system tailored for HAR in indoor flexible orientation settings, complemented by a meticulously designed identification procedure for HAR. Notably, to counteract the self-occlusion challenge endemic to flexible orientations, we integrate an indoor localization procedure and an adaptive weight adjustment mechanism. This system dynamically modifies its behavior based on real-time localization findings, harnessing the dual Kinect V2 system’s strengths and mitigating the adverse effects of self-occlusion.
(2) In our adaptive weight adjustment mechanism, some other factors may appear to bring us negative impact when we are introducing the indoor localization module. Therefore, some effective measures should be taken. For acoustic signals used in the indoor localization process and the NLOS transmission paths in real-world situations, a novel method based on a fuzzy c-means algorithm is introduced to optimize the Support Vector Machine (SVM). We treat it as a weak classifier. The amalgamation of multiple SVMs culminates in the employment of an enhanced AdaBoost as a potent classifier, proficient in discerning NLOS acoustic signals in dynamic settings. It helps this process to obtain a more accurate indoor localization result. Then, the localization process is completed based on the identification results. This aspect will help to assist the dual Kinect V2 system in completing the adaptive weight adjustment mechanism based on the indoor localization results.
(3) We present a cutting-edge feature extraction method based on skeleton joint data for identifying sitting, standing, raising one’s hand, and falling in tangible settings. Our HAR dataset with flexible orientations was produced. The Random Forest (RF) model is at the crux of this methodology. Addressing its inherent susceptibility to entrapment in local minima and its suboptimal parameter optimization efficiency—both of which compromise classification prowess—we propose a bat algorithm-optimized RF. Our approach greatly improves classification efficiency. Finally, we achieve extremely high HAR accuracy with the aid of an adaptive weight adjustment mechanism in indoor flexible orientation scenarios.

The arrangement of the remaining part in this manuscript is as follows: Section 2 provides an explanation of the core devices and the core theoretical methods used to complete the HAR task. The system framework and core adaptive optimization methods for flexible orientation scenarios are explained in Section 3. To make the methods in Section 3 more effective, Section 4 proposes our updating strategies based on different ensemble learning methods for the NLOS acoustic signal identification process and for the HAR process in the flexible orientation scenarios. Section 5 outlines the integration of the core method in our dual Kinect V2 system and compares our methods with other works in real-world situations. Section 6 provides a summary. Finally, the analysis for future works is revealed.

## 2. Related Devices and Methods

### 2.1. Kinect Devices

For the HAR task in indoor scenarios, systems utilizing three-dimensional data are pivotal. The classic approaches often rely on RGB images, point cloud models, or video streams. They are quite different from the skeleton joint data [1,3,7,15,21]. By comparing the joint data obtained through Kinect devices, AlphaPose (Shanghai Jiao Tong University, Machine Vision and Intelligence Group(MVIG), Shanghai City, China), OpenPose (v1.6.0), and other ways, as well as their core processes [7,10,13,18,19,20,28], it can be observed that joint data have some advantages in the HAR task. Firstly, user privacy is widely protected due to the special storage approaches in some situations. Secondly, joint data are less affected by darkness, light, and shadow conditions as they are obtained through infrared equipment, making them more suitable for complex indoor situations. Furthermore, it should be emphasized that the joint data can be smartly handled according to the SDKs in the Kinect devices, which provides great convenience for multimodal cross-platform interactive development objectively. The official SDKs are composed of various mainstream computer languages, such as C#, C++, and Visual Basic (VB). They represent a solid foundation and have been laid for those cross-platform interactive projects. By introducing engines like Unity Engine(2021.2.19f1) and Unreal Engine (v5.3), core scripts can be implemented based on the SDK, enabling cross-platform interaction and multimodal applications. Furthermore, it is smart to achieve interaction between multiple different types of devices within the same engine [17,36], which is an advantage that some online Application Programming Interface (API)s may not currently have. The Kinect V2 device has similar functionality compared to the Microsoft Kinect and Azure Kinect, which are of the same origin and clan. It is important to make reasonable choices when in different scenarios [12,13,18,19].

Scholars from various countries have conducted extensive discussions and research on the HAR task. They are intended to focus more on the joint data, depth data, or other visual information, with less emphasis on acoustic signals due to challenges like environmental noise, interference from communication devices, and materials in contact. These factors make it challenging to collect effective acoustic signals during transmission in complex scenarios. On the contrary, visual information is more straightforward and makes a direct attack on it. Consequently, many researchers have built various and colorful sensor networks based on skeleton joint data, deep data streams, or RGB streams. A variety of datasets have been constructed for HAR tasks, such as NTU-60, NTU-120 [31,32], UR-FALL [37], CMD FALL [38], Fall Dataset [39], PKU MMD [40], and UP-FALL [41]. Some other works have also built their datasets to complete the necessary identifications [7,13,14,20]. However, the dataset mentioned above may have nonnegligible limitations in flexible orientation scenarios. They have tight restrictions on the position of the user and the angle formed between the user and the camera in the real-world situation. These constraints expose the inadequacies of monocular systems, particularly their inability to address self-occlusion issues arising from dynamic orientations. It also greatly limits the practical effectiveness of identification methods, which makes it difficult to promote the core identification method in complex and flexible scenarios. To overcome these challenges, we propose a system that uses two Kinect V2s simultaneously, with wireless communication between the computers connected to each Kinect V2 accomplished through an improved TCP in a binocular system. To combat the adverse effects of self-occlusion scenarios on identification methods and improve the effectiveness of HAR methods in such scenarios, an adaptive weight adjustment mechanism based on indoor localization results is intended to be proposed. Additionally, this is bolstered by the integration of core ensemble learning techniques, ensuring a more resilient HAR methodology across various facets.

Upon analyzing the previously discussed works, several discernible trends emerge. To begin with, Kinect devices invariably come equipped with microphone arrays. The array it contains may be a non-uniform linear microphone array (such as Kinect V1, Kinect V2), or the microphones may be arranged in a benzene ring shape on the same plane (such as Azure Kinect). Although they have some limitations, they expand the feasibility of multimodal data development objectively [4,5,10,17,18,19,20], particularly for projects with high-precision skeleton joint tracking demands. Secondly, as for the projects on non-contact devices, researchers are more willing to be absorbed into the visual information, such as joint data, RGB data, and depth map data, rather than auditory information. This not only stems from the limitations of the device itself but also from the demands of the application site. On the one hand, audio signals are rarely used directly to complete interactive processes or HAR tasks in related works. On the other hand, opting for non-contact devices is not without its drawbacks. In the realm of visual data, there is ongoing debate surrounding the efficacy, affordance and appropriateness of RGB and depth map data in certain contexts [11,17,18,19,20,21], which may reduce the user experience sharply. As a result, skeleton joint data have witnessed a surge in worldwide interest and applications.

### 2.2. Ensemble Learning Method

The Bagging technique stands as one of the earliest formulations in the ensemble learning domain [42]. This method involves sampling with replacement from the whole dataset to divide it into different training subsets. For each subset, the base model (or base learner) completes training one by one. Subsequently, an amalgamation is executed, relying on distinct methodologies and tenets. A pivotal facet of the Bagging technique lies in instituting parallel processing for the base model within the primary procedure. This configuration permits the harnessing of collective wisdom by assimilating insights from various models. As a result, the classifier witnesses enhancement, particularly when deployed on imbalanced datasets. This facilitates a swift progression towards classification or regression analysis within random feature subspaces [7,42,43,44,45,46]. Among them, the RF model consists of Decision Tree (DT). It is adopted in many different applications. Some key parts such as preprocessing, splitting, bootstrapping, and weak classifier evaluation indicators should be re-designed wisely when implementing this model in our dual Kinect V2 system.

**Figure 2:**
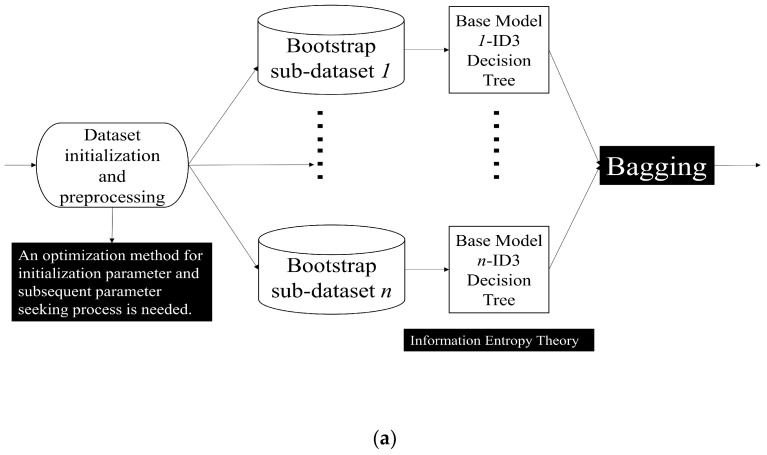

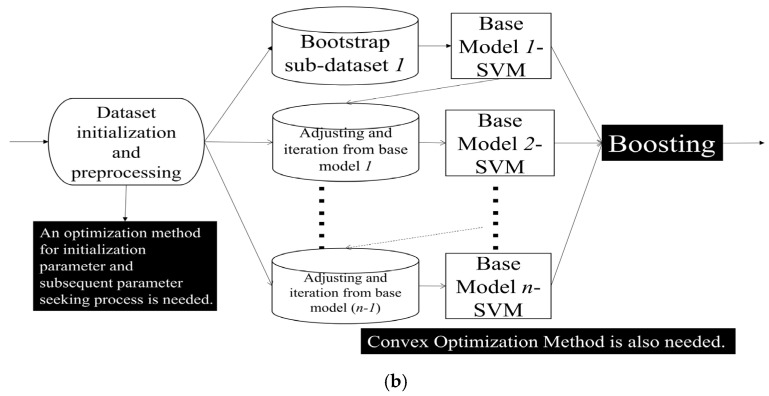
Key Ensemble Learning Method (**a**) Bagging Ensemble Learning Method (**b**) Boosting Ensemble Learning Method.

Boosting represents a distinctive ensemble learning approach, underscored by its iterative nature [7,47,48,49]. For each base model, identification results will be obtained for different subsets during the training process. For existing identification samples, after being processed by adaptive weight adjustment approaches, the weight of such samples may be appropriately increased and placed in the next base model to complete the next process. Iterative weighting and model training persist until either a predefined number of models are trained or the error rate plateaus. For AdaBoost and other models, such as Fair-AdaBoost [48], and SpatialBoost [49], those samples with poor training effects are supposed to be given more training weights. This intentional focus on harder-to-classify samples stands at the heart of the iterative improvement observed in Boosting algorithms. These processes will be described in Section 4 of this manuscript, corresponding to different modules in our dual Kinect V2 system. Our two ensemble learning processes adopted are as follows (Figure 2):

In our adaptive weight adjustment mechanism, some other factors may appear to bring us negative impact when we are introducing the indoor localization process. On the one hand, to assist in the mechanism, it turns out to be tremendous to cope with the NLOS transmission path issues. As for the Chirp (Linear Frequency Modulation, LFM) signals in indoor localization systems, it can be clearly found that the Chirp signal used in the system does not bring extra environmental noise and is beneficial for improving the filtering effect due to its significant difference in frequency distribution in the real-world situation [33,34]. As for each acoustic anchor, we consult some conclusions in [33,50]. However, traditional machine learning models may not perform well in complex or flexible situations. Therefore, we determined to use SVM as a weak classifier, and the Boosting method was adopted to construct an advanced AdaBoost method, which can complete the acoustic signal identifications and then improve the positioning accuracy. On the other hand, we decide to use ID3 DT as a weak classifier and use improved Bagging method to form RF and complete the HAR task in our system. To address the parameter-seeking issues, low classification efficiency, and lack of accuracy, an improved RF is proposed to complete the identification between the four common actions and their interference actions introduced by us in the classroom situation. We are quite convinced that the combination of the two modules mentioned above will enable the system to achieve more accurate HAR, especially in flexible orientation scenarios.

## 3. System Setup and Framework

### 3.1. Dual Kinect V2 System

**Figure 3:**
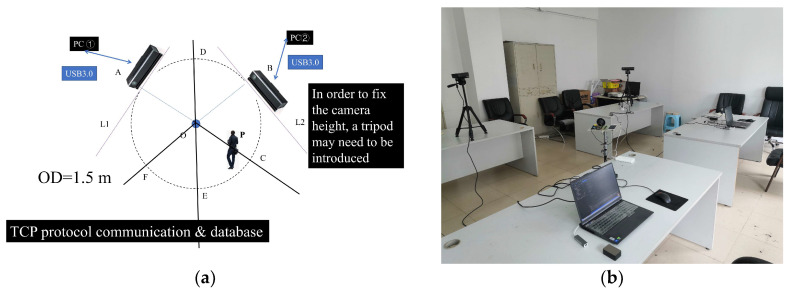
Dual Kinect V2 System Deployment Plan (**a**) Deploying Plan Model (**b**) Deployment in the Real-World Situation.

**Figure 4:**
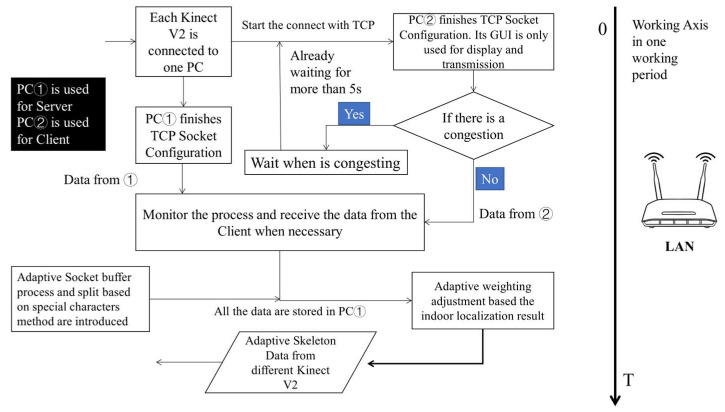
Improved Communication Process for Dual Kinect V2 System.

While certain algorithms have demonstrated notable efficacy, particularly for monocular Kinect systems, their applicability tends to diminish in intricate, dynamic environments. The orientation range is unfavorable to skeleton joint data and may not be suitable in some flexible orientation scenarios. Consequently, there is an increasing focus on multi-vision systems. For the Kinect V2, the interaction method among various PCs in the C/S (Client/Server) model can be implemented by using an improved TCP. Each Kinect V2 is bound to be connected to each PC one by one due to the facility principles and USB transmission speed limitations. Then, a multi-vision system will be built up for multiple Kinect V2s in the same network environment. Such a methodology is designed to mediate interactions of dual Kinect V2 configurations in wireless networks. Yet, unresolved issues persist. On the one hand, although some systems have already found orientation limitations in real-world situations [7,10,13,20], all those negative factors may have to be avoided to alleviate the self-occlusion issues consciously. Still, these observations merely skirt around the self-occlusion problem rather than addressing its root cause. As a matter of fact, if the system is directly built up according to the regulations of the 3D rectangular coordinate system under this circumstance, the ideal results may not be obtained. Our experiment results show that it seems to labor hard to little avail and has poor results. On the other hand, deploying the feature extraction algorithm constructed with joint coordinates in such scenarios does not avoid self-occlusion issues due to orientation ranges and inherent limitations of Kinect V2. This further exacerbates negative impacts. Using fixed headsets or other equipment to carry the device for an extended period is not ideal due to the weight and volume of Kinect V2 [18,19,20,21,22]. This will also lose the advantage of non-contact devices [11,12,13,14,18,19,20]. In light of these challenges, we introduce a novel approach termed the “tangent to the virtual ring” method, which will be detailed as follows (Figure 3):

∠AOB=90° in Figure 3a. We complete the analysis by referring to the computer network Open System Interconnection (OSI) seven-layer model. During each communication period, consecutive messages from the application layer might not be distinguishable in the buffering zone, given the limitations of protocols like TCP. This presents a challenge: the receiving end cannot confidently ascertain the completeness of information within current TCP segments, leading to the TCP Stick Package issue. To alleviate this, the User Datagram Protocol (UDP) may be directly used to complete the interaction in some systems. It has a certain positive impact indeed. However, as a classic unreliable and unstable transmission protocol, the UDP makes it difficult to avoid the risk of undetected events caused by packet loss. Especially for elderly users or some students, their actions are more unexpected and unpredictable in the real-world situation. Therefore, if all the IoT devices are linked in the same network environment, some measures are supposed to be taken. We also introduce specific markers to demarcate data after their collection and preprocessing for each Kinect V2. This entails mandatory segmentation based on the identified markers. At the same time, it is crucial to implement strategic enhancements during the interaction process within the C/S model, especially to manage potential network congestion. The long arrow on the right side represents the timeline. Its main workflow is as follows (Figure 4):

As for PC No. ① and the Kinect V2 it is connected to, its most suitable zone is $\overset{⏜}{\mathrm{COE}}$ in Figure 3a. According to the geometric relationships, for PC No. ② and the Kinect V2 it is connected to, its most suitable zone is $\overset{⏜}{\mathrm{EOF}}$. To avoid unnecessary self-occlusion issues and NLOS transmission paths as much as possible, it is essential to guide users into the required zone promptly. Before that, they are expected to have the ability to move and are willing to cooperate with the system’s process. Moreover, the system will adaptively adjust the data based on the results obtained by the indoor localization module in our system. It is anticipated that users will position themselves such that at least one Kinect V2 captures data from an optimal perspective, enhancing HAR tasks. Although each Kinect V2 may not always have a positive impact from an improper orientation, the data it receives ought to be an indispensable part of the identification method and not be completely ignored. If the user is located at P point, an adaptive weight-adjusting formula is introduced for the data received by each Kinect V2. For the identification process, each Kinect V2 needs to measure the direct linear distance from the geometric center of the camera to the Spine joint at present. Priority is then given to the device recording a greater distance. The indoor localization results will be used to calculate weights in the effective zone based on the area. The adaptive weight-adjusting formula for Kinect V2 at the far end is as follows:

(1) $$W_{far-end}=\frac{S_{\overset{⏜}{POD}}}{\frac{1}{2}\cdot S_{\Theta O}}$$

When a device registers a significant number of erroneous skeleton joint connections, resulting in substantial joint fluctuations within two seconds, data from an alternative Kinect V2 device will be favored. This rule will help the system shape the orientation before weighting. This guideline aids the system in determining the orientation prior to weight assignment. For users whose Spine joints are proximate to the camera within the effective range, their data should not be assigned higher weights. If the user has already entered the effective zone, the inappropriate distance and orientation with Kinect V2 will encounter a higher probability of suffering self-occlusion issues. The two Kinect V2 devices will greatly improve the identification performance while ensuring that at least one of them rarely has a negative impact on improper orientations. Integrating this with Formula (1), the adaptive weight-adjusting formula at the near end is as follows:

(2) $$W_{near-end}=1-\frac{S_{\overset{⏜}{POD}}}{\frac{1}{2}\cdot S_{\Theta O}}$$

**Figure 5:**
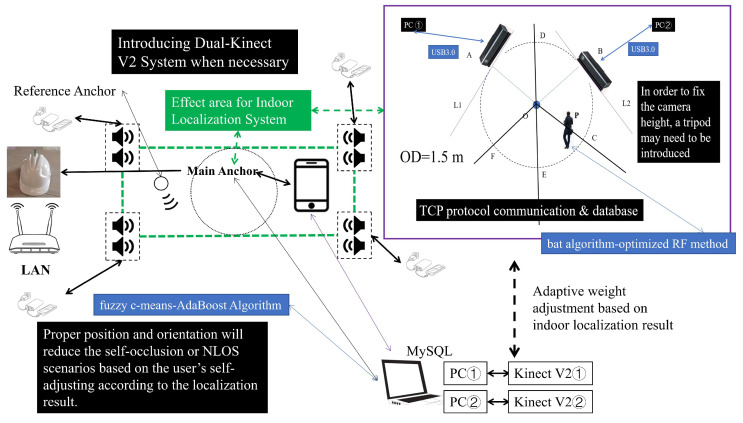
The Overall Framework of the System with Adaptive Adjustment Function.

The computation of weights is contingent upon user coordinates within the virtual ring, a foundational requisite for executing the aforementioned coordinates-based adaptive weight-adjusting mechanism. The conversion from coordinates to weights is obtained through the combination of coordinates and radians using geometric principles. The indoor localization system from [33,34,50] can be adopted and it will provide the localization results required for the dual Kinect V2 system. In certain instances, it may be necessary to affix or stabilize some IoT devices [33]. For complex scenarios, the incorporation of a dual-receiving structure is also posited [34]. Meanwhile, the Android smartphones linked to the acoustic anchors ought to install an arranged Android APP timely to control the localization periods. Micro-Electro-Mechanical System (MEMS) microphones can also be introduced to achieve necessary interaction with various acoustic anchors. The core circuit of the anchors and other modules required for the head-worn microphone can be referred to [33,34,51,52,53]. In our system, users have the authority to decide whether to use the indoor localization process and the adaptive weight-adjusting mechanism or not. The indoor localization module should cover the entire zone in a dual Kinect V2 system [33]. Users can employ headphones for interaction when deemed necessary. The indoor localization module is highlighted with a green dashed line, while the action recognition module, which relies on the dual Kinect V2, is marked with a purple rectangular box on the right side of the figure. The blue rectangular box’s text is appropriately set to explain the core methods or devices in the corresponding module. The effective working area is indicated by the black dot circle corresponding to the Main Anchor. The two modules are closely interrelated and inseparable. Different numbers are used to represent different devices. According to Figure 3a,b mentioned before, the framework of the system is as follows (Figure 5):

In reality, while each part of our system operates, they are interdependent and cannot be entirely isolated from one another. Through mutual collaboration between the two core modules, our system can effectively complete HAR tasks in flexible orientation scenarios without relying on high-complexity models. As previously noted, the indoor localization process serves as the foundation of the adaptive weight adjustment mechanism in our dual Kinect V2 system. It is worth noting that the system cannot achieve the expected results if it solely relies on the HAR method presented in this manuscript. Therefore, it is vital to make necessary modifications and maintenance during the overall deployment process of the system, particularly about the equipment used for the indoor localization process. Some indoor localization systems based on Chirp signals and Time Difference of Arrival (TDoA) methods can obtain significant results in the indoor real-world situation [33,34]. They may have the advantage of requiring less coverage in indoor scenarios, encountering fewer unexpected interference factors, and having a regular distribution of acoustic signals in indoor classrooms, meeting rooms, and other scenarios. These indoor localization systems based on Chirp acoustic signals, Ultra-Wide Band technology, and others can achieve higher positioning accuracy compared to the system outside based on Global Positioning System (GPS), Location Based Services (LBS), and other technologies in the real-world situation. In addition, indoor localization systems may also have lower power consumption and cost, making them an attractive option. The power supply equipment and network environments are also more stable. This is more in line with the demands of our dual Kinect V2 system. Furthermore, GPS, LBS, etc., cannot be fully used normally due to the obstruction of the building shells. Under the comprehensive influence of the above factors, our system is expected to be deployed and used in indoor situations.

### 3.2. Preprocessing and Feature Extraction Method

**Figure 6:**
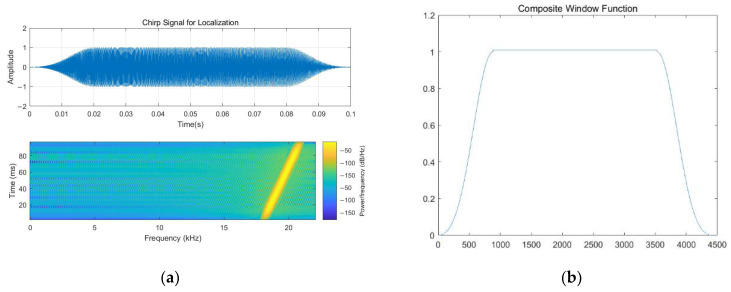
The signal modulation process and the composite function (**a**) The Chirp Signal after being Processed (**b**) The Composite Window Function.

**Figure 7:**
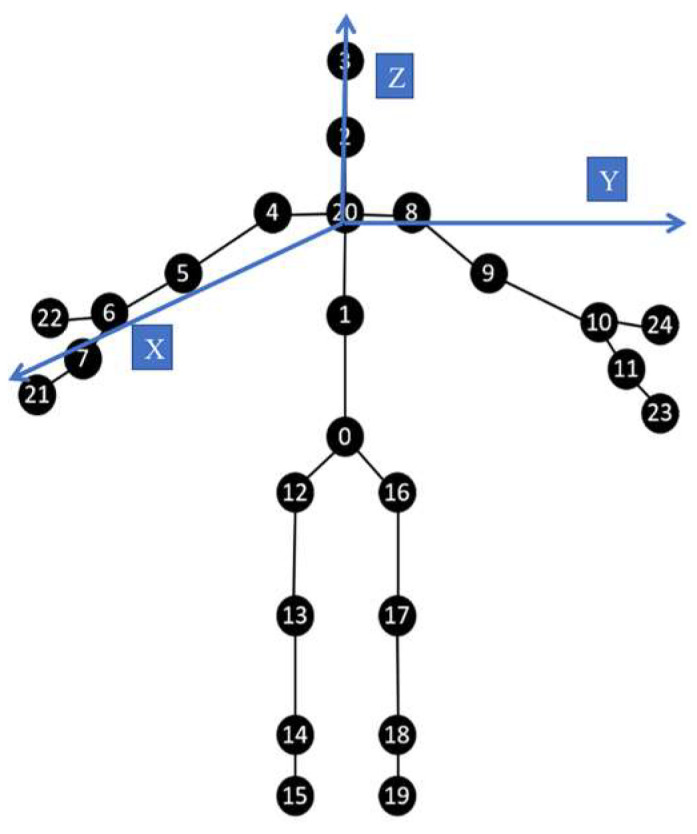
Kinect V2 joints.

In intricate scenarios, the indoor localization system augments the HAR task by integrating both the NLOS acoustic signal identification technique and the subsequent processing steps [33]. On the one hand, as for the indoor localization module, the frequency band used for the acoustic signal is designed according to [33,34,50], which is set to 18.5–20.5 kHz. Notably, there exists a distinct frequency distribution disparity between Chirp signals and indoor environmental noise. A composite window function is needed to complete the modulation when necessary before the Chirp acoustic signal is received by the terminal. The composite function, as described in [33,34], consists of a Blackman window and a rectangular window. Among them, the Blackman Window is cut into two pieces on average. Then, two pieces are placed on both sides. As noted in [33,50], the sampling rate of the Chirp acoustic signal is 44,100 Hz, and the default amplitude value is set to 1. The remaining component will be adsorbed onto the rectangular window. At the same time, additive white Gaussian noise (AWGN) can also be appropriately added to improve the robustness. They can be shown as follows (Figure 6):

On the other hand, the skeleton joint data might inadvertently introduce noise due to involuntary oscillations or tremors. For feature extraction algorithms predicated on 3D spatial joint coordinates—such as those from Leap Motion and Kinect devices—extraneous noise adversely affects performance. A principal joint, when connected to other joints, yields a dispersion pattern in spatial noise distribution. This compromises the efficacy of the feature extraction process. Consequently, the feature extraction method should prioritize the joint with the minimal tracking error. For instance, the Palm Center joint in the Leap Motion system is often used in the interactive gesture feature extraction process. The fundamental reason is that the tracking accuracy of this joint in the Leap Motion system is relatively stable, and large-scale fluctuations do not often appear [14,36,52]. Similarly, we suggest that the feature extraction process for Kinect V2 should focus on the Spine joint in complex situations. Moreover, we introduced a sixth-order Butterworth filter for the preprocessing process in the system. Its passband cutoff frequency is set to 2 Hz, the sampling frequency is set to 15 Hz, the maximum passband attenuation is set to 1 dB, and the maximum stopband attenuation is set to 40 dB to filter out unwanted parts in the dual Kinect V2 system. Numbers here are used to represent different skeleton joints. The tracking skeleton joints in Kinect V2 can be shown as follows (Figure 7):

Subsequently, a novel feature extraction algorithm will be proposed based on 3D coordinates. Drawing on Ergonomics, Inverse Kinematic theory, and mirror symmetry relationships, the feature algorithms will be developed. For the 3D coordinate system in Figure 7, we make the plane XOY parallel to the ground, and the Spine Shoulder is used as the O point, with a vertical upward direction as the *Z*-axis direction. The vertical plane of the user’s right arm is constructed in the *X*-axis positive direction, and the left arm of the trunk is oriented in the *Y*-axis positive direction to establish a coordinate system. The feature extraction algorithm used mainly consists of the following three aspects:

① First, the distances from the joints to each plane in the 3D coordinate system is taken. The distances to XOY, XOZ, and YOZ from Wrist Left, Elbow Left, Knee Left, Ankle Left and their symmetrical joints Wrist Right, Elbow Right, Knee Right, and Ankle Right are both being considered. The results obtained will provide key feedback on the changes in static features, but the effect is not quite promising for dynamic features [20]. Therefore, other types of features are also imperative.

② In order to balance the feature set corresponding to some drastic and sharp actions in HAR tasks, some joints are selected to complete feature extraction by calculating the angle built by three selected joints in one group [18,19]. As for the set S contained by selected joints, referring to the indexes from Figure 7, all these joint index numbers in the set S can be written as $S=\left\{\left.\left[1,8,9],[1,8,10],[1,16,17],[1,16,19],[1,4,5],[1,4,6],[1,12,13],[1,12,15\right]\right\}\right.$. Each three skeleton joints contained in the every matrix from S are adopted to construct all the angles. Then, the angle is calculated based on the mathematical principles of vector computing. The Spine Mid, the Shoulder Right and the Elbow Right are taken as an example, and these joints are represented as A, B and C here. After obtaining the coordinates in the dual Kinect V2 system, if $\vec{a}=\vec{BA}$ and $\vec{b}=\vec{BC}$ is required, ∠ABC can be gotten from:

(3) $$∠ABC=\arccos\left(\frac{\vec{a}•\vec{b}}{\left|\vec{a}\right|\cdot \left|\vec{b}\right|}\right)=\arccos\left(\frac{x_{1}x_{2}+y_{1}y_{2}+z_{1}z_{2}}{\sqrt{x_{1}^{2}+y_{1}^{2}+z_{1}^{2}}\cdot \sqrt{x_{2}^{2}+y_{2}^{2}+z_{2}^{2}}}\right)$$

③ Although the approach in ② is commonly used in feature extraction algorithms for skeleton joint data, it still has some limitations. For fixed perspective and static feature sets, it can dynamically feedback on subtle changes in the HAR task. However, as for the dynamic feature sets from different orientations, the above features still have restrictions on sharp actions. The feature extraction algorithms in unsuitable angles lack robustness. They may often be influenced by the noise and accidental vibration [51,52,53]. Meanwhile, we introduce the dynamic triangle area method to construct new features from ②. As for the set S, we calculate the area of triangles formed by joint data in each group separately. Considering the influence of the orientations and distances in the real-world situation, it does not mean that the larger the angles, the larger the triangle area. Therefore, we believe that this feature will further highlight the dynamic parts. Taking the Spine Mid, Shoulder Right, and Elbow Right as examples, if they are represented by A, B and C sequentially, the following formula can be obtained:

(4) $$S△ABC=\frac{1}{2}\cdot \left|\vec{a}\right|\cdot \left|\vec{b}\right|\cdot \sin∠ABC$$

It should be noted that the triangle mentioned above does not always be parallel to YOZ. This will not lead to harmful influence in most situations. There are 40 kinds of features in total. Different from the joint feature extraction methods in monocular systems, traditional static threshold-based identification methods cannot cope with complex situations from multiple orientations [20]. Therefore, we determined to introduce the three components mentioned above simultaneously. While this augments the system’s complexity, we believe the trade-off is justified.

## 4. Optimization Strategies of Improved Ensemble Learning Method

### 4.1. NLOS Acoustic Signal Identification Based on Fuzzy C-Means Algorithm and AdaBoost

For the indoor localization aspect of the dual Kinect V2 system, it is vital to implement the feature extraction algorithms of Chirp acoustic signals in the system and complete the identification of acoustic localization signals in complex NLOS scenarios. Broadly, there are two primary approaches to addressing NLOS transmission paths for various acoustic anchors. The first approach involves identifying NLOS acoustic signals and then removing or eliminating them in some situations [33,34]. The second is to find the NLOS acoustic signal and finish compensating for energy loss or other losses [50]. Given that these losses in intricate scenarios are erratic and unmanageable, and considering the substantial difference in physical characteristics of signals in LOS versus NLOS conditions, our approach leans towards the first strategy for TDoA algorithm. This decision is rooted in addressing the NLOS acoustic signal challenges within the TDoA algorithm. Many works have completed the NLOS acoustic signals identification based on models such as logistic regression [50], improved SVM [33], and improved DT [34], and obtained excellent results in some situations. However, the classification methods are constrained by the convex optimization performance in complex situations, resulting in poor results [33,34]. Therefore, we propose an improved Bagging approach based on the process in Figure 2a to address this issue. Subsequently, a sophisticated AdaBoost comprising SVM elements will be developed for NLOS acoustic signal identification, aiming to bolster the positioning accuracy in the dual Kinect V2 system. Simulations of NLOS conditions draw inspiration from the research presented in [33,34,50]. To overcome the limitations of weak classifiers and low training efficiency, multiple weak classifiers are assembled, and an adaptive iterative process is adopted to construct an advanced AdaBoost with stronger learning ability. Given the drawbacks of inadequate convex optimization capabilities and inefficient training, we combine multiple weak classifiers. This amalgamation, together with an adaptive iterative process, crafts an enhanced AdaBoost possessing robust learning competencies. A novel adaptive methodology, grounded in the fuzzy c-means algorithm, is proposed to refine AdaBoost, thereby amplifying its identification capabilities. Within this system, nine weak SVM classifiers are amalgamated, and the NLOS acoustic signal identification outcomes are presented in each indoor localization loop. The novel fuzzy c-means-AdaBoost algorithm for the NLOS acoustic signal identification method follows a specific process:

*Stage 1*: As for each acoustic signal feature, it can be seen as a single, non-linearly independent set of spatiotemporal samples. If there are 2n samples in total, we can obtain $\left(x_{1},y_{1}),(x_{2},y_{2}),\ldots ,(x_{2n},y_{2n}\right)$. 10,000 Chirp signal samples are used in total, 5000 for LOS acoustic signals and 5000 for NLOS acoustic signals.
*Stage 2*: Before training each weak classifier SVM, the weight of the i-th group training can be recorded as: $w_{i}=\frac{1}{2n}$, i=1,2,…,2n. The initial weight should be set as $w_{1,x}=w_{x}$. In T-th iteration process, we normalize each weak classifier SVM initial weights separately and then adjust the adaptive weights based on the dynamic threshold. The algorithm pseudocode can be described as (Algorithm 1):
**Algorithm 1.** Adaptive Weight Adjustment Method for AdaBoostFor t = 1:T{$q_{t,j}=\frac{w_{t,j}}{\sum_{y=1}^{2n}w_{t,y}}$/*Normalizing the weights of each weak classifiers *//*Each identification result for weak classifiers needs to be recorded as $h{\left(d\right)}_{t,j}$*//* The adaptive error rate of the m-th classifier needs to be calculated*/    For m = 1:M      {/*Finding out the lowest adaptive error rate */        $\varepsilon_{m}=\sum_{i}[q_{t,j}•h{\left(d\right)}_{t,j}]$        $\varepsilon_{least}=\min(\varepsilon_{1},\varepsilon_{2},\ldots ,\varepsilon_{m})$        $w_{t+1,i}=w_{t,i}\beta_{i}^{l_{m}}$, $\beta_{i}=\frac{\varepsilon_{least}}{1-\varepsilon_{least}}$       }    End For/*l is used for the label, l=1 means the misclassification, l=0 means correct classification */}End For
*Stage 3*: The results can be obtained through the previous iterative process and we are able to complete the weight determination process in the ensemble learning method. Meanwhile, the decision function in the advanced AdaBoost classifier can be written as:

(5) $$C(x)=\left\{\begin{array}{cc} 1 & \sum_{i=1}^{T}\alpha_{d}•h_{i}(x)\geq s•\sum_{i=1}^{T}\alpha_{d}\\ 0 & else \end{array}\right.$$

$h_{i}(x)$ represents the determination result obtained temporarily, s is an auxiliary static threshold. We set s=1/2. After updating the decision function in the system, the overall result can be gained through the adaptive adjustment process. Furthermore, this classifier property yields good generalization performance and obtains an extraordinary convex optimization ability without introducing excessive complexity. In this way, it upgrades the performances in complex scenarios. Furthermore, it is possible to assist the dual Kinect V2 system in HAR tasks in flexible orientation scenarios if the users are willing to obey the rules. Finally, the system may need to follow the approaches in [33] to fix the relevant acoustic anchors in a suitable place when necessary, which will greatly reduce the unnecessary and unexpected NLOS transmission paths.

### 4.2. Bat Algorithm-Optimized RF Method on HAR Task

Traditional machine learning models are gradually exposing their limitations. Multi-classification models require sufficient convex optimization capabilities when adopting appropriate preprocessing and feature extraction methods. However, if parameter optimization in these models is not adeptly managed, there can be excessive time spent on model parameter tuning, compromising real-time and accurate identification. Consequently, some actions in the HAR task may be susceptible to misclassification during certain periods, leading to the weight of those misclassification weak classifiers being continuously increased. Moreover, improperly configured normalization factors can elongate training durations. Therefore, optimization methods based on some swarm intelligence algorithms [33], Gravitational Search Algorithms [34], and other models are playing a tremendous role in various fields [3,4,5,6,7,8,9,10,11,12,13,14,15,20,22,36,37]. To address these issues, we focus on the skeleton joint data system and complete the identification through the Bagging method in the dual Kinect V2. The parallel processing ability attaches great importance to our system. Based on the work in [34], we consider DT as a weak classifier, while optimizing the RF model by using the bat algorithm. Last but not least, combined with the adaptive weight adjustment mechanism, the identification of sitting, standing, raising one’s hands, and falling action databases including their interference actions will be completed.

The bat algorithm is a heuristic optimization algorithm. It introduces a proper optimization mechanism and efficient parameter searching process to improve the performance of some traditional machine learning models [54]. In this way, further enhancements are obtained, and the adaptive strong classifier is promoted. In the algorithm, parameter optimization in the ensemble learning model is achieved by simulating the hunting process of biological bats. Among them, the algorithm assumes that these bats send electromagnetic waves at a fixed frequency $f_{i}$ in the initialization process, during which they fly at speed $v_{i}$ in $x_{i}$ and complete the preying process. They track the approximate prey’s position through the principle of echolocation extensively. They can also adjust the signal frequency, wavelength, and loudness intime while their targets are lurking. Compared with traditional swarm intelligence algorithms such as Genetic Algorithm, Particle Swarm Optimization, and so on, this algorithm will simultaneously perform global and local searching during the optimization process, which can further avoid the possibility of falling into local minimum regions and accelerate the training [54]. This is particularly evident in ensemble learning methods. It also emphasizes parallel computation performances. In our method, the bat algorithm can transform the parameter-seeking process into an optimization process with a single objective function and slack the constraint conditions. In the initial phase, the sample set can be written as $X_{i}^{0}=\{x_{i,1},x_{i,2},\ldots ,x_{i,n}|i=1,2,\ldots ,M\}$. n represents the dimension of feature sets. M represents the number of infantries in the population. The range of the infantries during the training process can be written as follows:

(6) $$x_{ij}=L_{j}+rand(U_{j}-L_{j})$$

Among them, $x_{ij}$ represents the positions of the infantry i during the j-th iteration process. $U_{j}$ and $L_{j}$ correspond to the upper and lower boundaries of the effective region, respectively. rand() is a random value from [0,1]. For the parameter optimization process, essential and needful updates or iterations are completed through the following formulations:

(7) $$f_{i}=f_{\min}+(f_{\max}-f_{\min})\cdot \beta ,\beta \in [0,1]$$

(8) $$V_{i}^{T}=V_{i}^{T-1}+(X_{i}^{T}-X^{*})\cdot f_{i}$$

(9) $$X_{i}^{T}=X_{i}^{T-1}+V_{i}^{T}$$

(10) $$X_{new}=X^{*}+\varepsilon {\bar{A}}^{T},\varepsilon \in [-1,1]$$

(11) $$A_{i}^{T}=\alpha •A_{i}^{T-1}$$

(12) $$r_{i}^{T}=r_{i}^{0}(1-e^{-\eta T})$$

β is considered a random variable. $f_{i}$ is the pulse frequency of the signal emitted by the i-th infantry. $f_{\max},f_{\min}$ are the maximum and minimum values of the transmitted signal frequency, respectively. $X^{*}$ is a tentative global optimal position. $X_{i}^{T}$ means the position at present. $V_{i}^{T}$ means the velocity at this moment. $A_{i}^{T}$ is the amplitude value of the signal emitted at this moment. ${\bar{A}}^{T}$ is the average amplitude value of the signal emitted by the bat. ε is a random number within a specific range. Once the bats discover their targets, the loudness of predators is gradually decreasing. We complete the simulation through (11). At the same time, the probability of pulse transmission $r_{i}^{T}$ also needs to be considered. $r_{i}^{0}$ is set to 0.75. α and η are constants within the range of [0,1].

We can optimize the RF by combining the methods in Figure 2b. Obviously, some parameters in RF directly determine the results of HAR tasks, such as the number of DT or estimators L, the sample split numbers m, the number of predicting samples Y, the pruning threshold θ and so on [46,47,54]. In order to improve the identification accuracy in the dual Kinect V2 system, the optimization process is upgraded according to the number of DT L and the sample split numbers m under this circumstance. They can be regarded as the guiding indicators for the optimization feedback. All these infantries are able to share historical experiences on finishing the global optimization and local optimization. The core optimization process is as follows:

*Stage 1*: Initialization. The maximum number of iterations in the algorithm is set to 200. Meanwhile, the number of the infantries M, the initial position of each infantry $x_{i}$ and its velocity $v_{i}$ should also be set. The parameters L and m in the process are used to illustrate the states of optimization method in subsequent iteration and updating process.
*Stage 2*: Assuming that the current position of the infantry is the global optimization position, the fitness value is recorded as $P_{best}$. They are brought into the RF to update the individual fitness function and the out of bag error $e_{odb}$.
*Stage 3*: The infantry compares the fitness value with the local optimization value, and selects the larger value as the target, which is recorded as $P_{g}$.
*Stage 4*: During each iteration, operation and iteration results need to be fed back in the algorithm, so as to update their positions and velocities.
*Stage 5*: The comparisons with $P_{g}$ and $P_{best}$ should be finished. When it comes to the maximum number of iterations or the $P_{best}$ is not changing, the optimization process can be terminated and the core process can step into *Stage 6*. Otherwise, the process should return to the *Stage 4*.
*Stage 6*: L and m are supposed to be outputted according to all these stages mentioned above. Then, the improved RF model is totally given on this basis, and it leads to a better performance on HAR task in dual Kinect V2 system.

**Figure 8:**
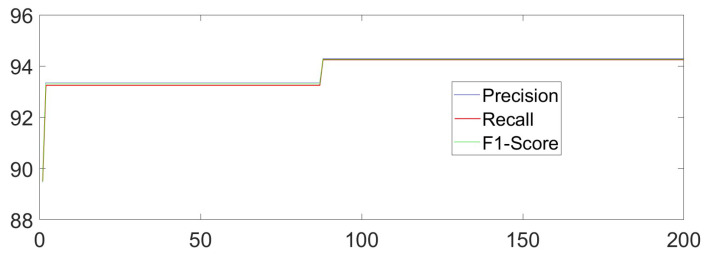
The Iteration Process.

The dataset is meticulously curated to accentuate the advantages of our primary methodology. MATLAB 2017a and Python 3.6.2 are adopted to implement our core methods. TensorFlow 1.14.0 and PyTorch 1.10.0 are chosen as the necessary deep learning tools in our experiment. The Unity Engine (2021.2.19f1) may also be used when necessary. The PC used in this manuscript is Lenovo Y9000P (from Lenovo Inc., Beijing, China) and Alienware ALWX15-R1978W (from Dell Inc., Round Rock, TX, USA), which has 11th Gen InteI) I(TM) i7-11800H, NVIDIA GeForce RTX 3060 GPU and RTX 3080 GPU, respectively. Building on this foundation, the bat algorithm or other necessary methods are implemented to optimize RF and the identification process for sitting, standing, raising one’s hands, and falling actions. To better adapt to flexible orientation scenarios, especially for the actions in classroom situations, the dataset we constructed introduced multiple interference actions one by one in different tasks. We added crossing one’s legs, seizing something on the desk, drinking actions, and ting one’s shoes to be interference actions for sitting, standing, raising one’s hands, and falling sequentially and separately. After adding four types of interference actions, 20,000 samples are randomly selected, with 5000 samples for each type of action. We randomly select 80% of the samples as the training set and use the remaining samples as the testing set. To comprehensively measure our methods by introducing the adaptive weight adjustment based on indoor localization results monolithically, we combine the formulas in [7,10,20] to calculate Accuracy, Precision, Recall, and F1-score. The overall recognition Accuracy of the HAR task is 94.25%, the Precision is 94.14%, the Recall is 94.00%, and the F1-score is 94.07% by using our own. The *X*-axis represents the number of iterations in training, while the *Y*-axis represents the value of the indicators. The overall iteration process is as follows (Figure 8):

Meanwhile, if there are not too many flexible orientation scenarios in the real-world situation, singular value decomposition (SVD) can be used without weighing the perspectives in some conditions. The feature contribution rate can be calculated according to the eigenvectors or other indexes [52,53]. The feature sets of HAR should be re-selected and re-arranged. At that moment, it will avoid the “disaster of dimensionality”.

## 5. Experiment and Analysis

Our experiments are divided into two sections. The first section is dedicated to exploring the identification methodology for NLOS acoustic localization signals, where we juxtapose actual indoor localization outcomes both before and after the application of our iterative optimization strategies. In the second section, we finish the HAR task according to the self-occlusion compensation mechanism. It consists of the indoor localization process and the adjusting mechanism upon it. We are steadfast in our belief that the cooperative interaction between these two facets will amplify the performance outcomes in HAR tasks. We reproduce some previous works and make appropriate modifications according to the requirements of the cross-platform interaction project. According to [33,34], more dynamic, flexible, and complex NLOS transmission paths are introduced into the experiments. The novel Chirp acoustic signal NLOS identification method is compared with other methods, as shown below (Table 1):

Our findings indicate that our proposed NLOS acoustic signal identification method excels in performance. The Boosting approach significantly enhances identification, harmonizing effectively with the NLOS acoustic signal mitigation strategy as highlighted in references [33,34]. Then, it greatly promotes the positioning accuracy of the system. The error calculations are finished one by one towards fixed coordinates. After adding the NLOS acoustic signal coping mechanism in [33], the average error of the obtained results is as follows (Meters) (Table 2):

The deployment of an indoor localization system using Kinect V2 and the Direction of Arrival (DoA) algorithm, while simple in concept, is significantly hindered by the angle range of the receiving terminals. And it is nearly impossible to achieve desirable positioning accuracy from a wider scope. The positioning error may be within 1m in a small range (e.g., 1 m × 1 m), and it is not suitable for the adaptive weight adjustment mechanism in our dual Kinect V2 system. Once the effective range is exceeded, such errors will rise sharply. Therefore, we have to abandon this system, and the indoor localization systems in [33,50] are regarded as the prototype. We believe that the refined fuzzy c-means-AdaBoost method serves better as an auxiliary element for our dual Kinect V2’s adaptive weight adjustment mechanism. In the flexible orientation scenario, our system conducts HAR tasks based on the indoor localization process, bolstering its resilience. If obstructions make skeleton joint data inaccessible, the HAR task in this loop will be terminated. The system is expected to wait until our system can collect all the data entirely, and complete the subsequent identification.

Our datasets are built in the real-world situation. For the actions to be identified, we invited some volunteers to assist us in completing the dataset production. The volunteers (9 males and 1 female) are both on an informed consent basis from participating in the experiments, which comply with the standards of the institutions. All the processes also comply with the local government’s standards and the entire scheme does not break any sensitive rules such as religion, military affairs, disease prevention principles, and so on. The angles between the front of the volunteers and the plane of any Kinect V2 camera needs to be controlled within [−135°,+135°]. In fact, this does not need to be deliberately controlled. Our dataset differs from other large-scale public datasets, such as NTU and UP-FALL, in that it places greater emphasis on the impact of flexible orientation scenarios on data collection. This distinction means that our dataset’s scope is not entirely the same as those of other large-scale public datasets. These public datasets with a large number of participants greatly benefit the robustness of HAR. However, they often involve fixed or pre-designed orientations during data collection, with sensors or cameras remaining stationary. It is unrealistic to refer to the production process of public datasets and complete actions from all possible angles in real-world situations and collect them one by one. Therefore, it is essential to use both our dataset and public datasets from multiple aspects in a dialectical manner. On the one hand, this will make it impossible to avoid or compensate for the negative impact caused by the connection mechanism of skeleton joints in some negative perspectives in the aforementioned dataset. On the other hand, for actions in most scenarios, if the impact of poor orientation is relatively small, these different kinds of actions and obtained skeleton joint data in large-scale datasets do have a significant influence on action recognition algorithms. There is no doubt that they can energize the algorithms.

**Figure 9:**
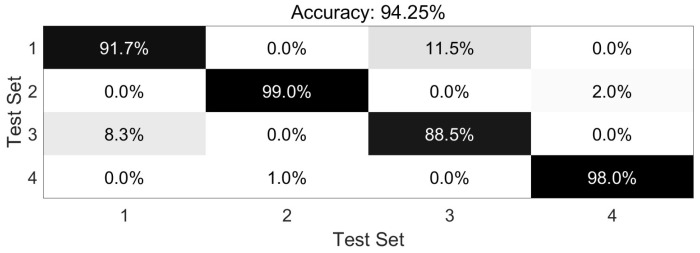
The Accuracy Comparisons of four HAR tasks.

It can be clearly seen that many previous studies focus on actions from a single perspective or a static position, where users act based on directives from administrators or medical experts, as seen in [1,7,10,11,12,13,16,20,35,37,46]. While effective in controlled settings, these methods often fall short in dynamic orientation scenarios, particularly when facing inherent self-occlusion challenges. To address this gap, we develop our system and implement our methods to perform experiments on four different action identification tasks in flexible orientation scenarios. Furthermore, the figure below will use the numbers 1-4 to represent four action recognition tasks: sitting, standing, raising one’s hands, and falling actions. Meanwhile, the interference actions will also mix among them appropriately. The darker the color, the higher the percentage. The accuracy comparison of the four tasks in total average is as follows (Figure 9):

According to the multi-classification task accuracy comparison results, the standing identification task and falling identification task have high accuracy and obtain promising results. Even in the indoor flexible orientation scenarios, it can be found that the interference actions added seem not to have an influential impact on the classification task. Some previous works have also demonstrated excellent results in fall and standing detection [7,10,13,14,20,35,37,38,39,40,41,45,46]. However, the identification effect of the sitting task is slightly less than that of the other two tasks. Although our novel methods are introduced and the final results are promoted according to the adaptive weight adjustment mechanism based on the indoor localization process, there are still occasionally wrong identification items.

We firmly believe that the two core modules of our system are not supposed to be separated. The indoor localization result is a crucial aspect of the adaptive weight adjustment mechanism in our system. If only our action recognition algorithm is utilized alone to complete the HAR task, the system’s performance potential cannot be fully realized. We evaluated our identification algorithm using NTU-60 and NTU-120 datasets, and compared it with some previously mentioned deep learning methods. Our evaluation focused on single-person scenarios, and only the four chosen actions mentioned above were tested in our dual Kinect V2 system. As for single-person scenarios, we observe that the public datasets mentioned above did not intentionally introduce interference actions in classroom situations. However, it is worth noting that deep learning algorithms have high requirements for computing. Therefore, we need a more reasonable design process for our dual Kinect V2 system. All tests here are run in offline mode. For different actions, each frame was processed during its working loop properly. The Unity engine also requires some additional settings due to the use of a GPU. We use the same dataset to compare our method with related works in [7,10,13,14,20,35]. If the adaptive weight adjustment mechanism based on indoor localization results is not introduced, the algorithm comparison results are as follows (Table 3):

Regarding the NTU-120 dataset, it encompasses a minimum of 120 distinct actions. The abundance of actions fostered a more profound comprehension of the system. Our comparison results, as illustrated in the table above, reveal that our methods are less effective than they ought to be without the introduction of adaptive weight adjustment mechanisms based on localization results, as compared to other deep learning models. Notably, whether it is the Chirp acoustic signal identification method based on ensemble learning in the dual Kinect V2 system or the c-means-RF method for skeleton joint data, the overall performance is inferior to that of some novel deep learning methods. Our improved ensemble learning method performs well in the standing and falling action tasks, but its efficacy in the raising one’s hand task is not as remarkable. We need to analyze the accuracy comparison results from multiple perspectives. On the one hand, the novel deep learning model demonstrates outstanding performance in the HAR tasks. This is attributable not only to the model’s strong understanding and learning abilities but also to the diverse and representative human action data in public datasets. Both the single-person scenarios and multi-person scenarios are included. On the other hand, the diversity in HAR tasks is not only reflected in the types of actions but also in the angles formed between the actions and the camera. The cameras located at fixed angles in these public datasets expose their drawbacks in the raising one’s hand task. In some unfavorable orientations, the feature extraction during the identification process becomes unstable and unpredictable due to the limitations of the skeleton joint connection mechanism, which is not conducive to HAR tasks in flexible orientation scenarios. Therefore, the GAN-based method may be used to generate partial skeleton data in some systems [28,29,30]. It cannot be denied that this can settle the self-occlusion issues to some extent, but it is evident that there is still a gap between the generated data and the actual data in real-world situations. We believe that introducing an adaptive weight adjustment mechanism based on the indoor localization process here is a more reasonable choice. Two core modules are supposed to work together. We completed a comparative experiment between our full version of the method and other key methods. The results obtained are as follows (Table 4):

According to our observations, some conclusions are coming out. First of all, those monocular systems on a fixed perspective are constructed in some previous works, and it can be seen that they have restricted the interaction zones a lot. During these situations, some interference actions, such as crossing one’s legs action in the sitting identification task, and drinking action in raising one’s hand task, increase the difficulty in these tasks. The limited orientations greatly lead to a huge loss in the practical identification methods, which are even more obvious in some tasks. In particular, the classification between raising one’s hand and drinking action is not so attractive in the flexible orientation scenarios. Our method boosted the effects in the dual Kinect V2 system compared with others, and the maximum improvement can reach about 30.25%. This suggests that while deep learning methods in fixed perspectives have outperformed our models, the introduction of an adaptive weight adjustment mechanism in flexible perspective scenarios is a more pragmatic approach. This is evidenced by the classification comparison in the raising one’s hand task, where our method incurs a smaller computational burden. Additionally, unlike some methods that focus on RGB video streams, our approach is suitable for both daytime and nighttime situations. Meanwhile, in fixed perspective scenarios, we cannot ignore the excellent results of the deep learning model. Based on the actual requirements of system deployment scenarios and hardware performance limitations, developers need to choose a more reasonable solution. Sometimes, some HAR tasks may still achieve outstanding results in monocular systems by using conventional machine learning or deep learning methods due to their good performances. In such conditions, it is not necessary to introduce the binocular theory to build up the system and consider other factors such as calculating and fixing Kinect V2 height, pitch angle, and so on. Another crucial distinction to make is between slipping and tripping behaviors in fall detection. Tripping may be easily confused with tying shoelaces in the real-world situation and this phenomenon can be usually found in our experiment. Some previous works are not considering these factors at present, which restrict the application scope of their methods under this circumstance [7,10,20,31,32,50,51,52,53,54]. Therefore, they may have to increase the types of data covering different orientations as much as possible in combination with the indoor complex situations. However, this approach can significantly increase the workload and may not meet the needs of all orientations. This also indicates that although the GAN-based method can generate some skeleton joint data for actions from a negative perspective required during the training process, its performance in real-world situations is obviously not as good as the real skeleton joint data that exists in weighted binocular systems. Therefore, we integrate the binocular system and the adaptive weight adjustment mechanism according to the indoor localization results. Our updating strategies on ensemble learning methods have also been adopted to carry out tremendous improvement and obtain excellent recognition ability. Last but not least, Kinect devices can also complete HAR tasks in a dark environment, which are beneficial for indoor complex situations. Given their ability to adapt to flexible orientation scenarios, HAR tasks in these scenarios are a more suitable choice.

## 6. Conclusions and Future Works

As for HAR tasks in complex indoor scenarios, the self-occlusion issues caused by flexible orientations will greatly decrease the effectiveness of the identification method. Therefore, we developed a dual Kinect V2 system. This system incorporates a novel HAR methodology grounded in various ensemble learning techniques. In our system, there are two core aspects. One is the HAR module, the other is the indoor localization module. These modules work in tandem, and our core method builds upon this foundation.

Firstly, we propose the “tangent to the virtual ring” method to develop our system based on the improved TCP and dual Kinect V2 devices. Additionally, an adaptive weight adjustment mechanism according to the user’s localization results is proposed. It can be seen that our dual Kinect V2 system deployment turns out to be more suitable for flexible orientation scenarios. Even when users are not in a proper orientation, the system can still obtain a convincing identification method based on the skeleton joints in time. To enhance our core methods, we present a novel feature extraction method with high persistence for the HAR tasks in classroom situations. Both indoor localization and HAR require thoughtful consideration in intricate scenarios. The indoor localization outcomes underpin our adaptive weight adjustment mechanism, though challenges may arise that necessitate effective countermeasures. Thus, some effective measures have to be taken. Then, different ensemble learning methods are used to cope with the following works. On the one hand, as for the NLOS transmission paths during the indoor localization process, the NLOS acoustic localization signal identification process in dual-receiving structures or flexible scenarios needs to be re-considered. Otherwise, they may bring a decline in positioning accuracy. Therefore, we propose a Chirp acoustic signal identification method by using fuzzy c-means-AdaBoost. The novel identification accuracy is 7.63% higher than the original AdaBoost method. This enables a more accurate NLOS compensation mechanism and reduces the final positioning error to less than 12cm in complex situations. The accurate localization result will become the key to the adaptive weight adjustment mechanism in our system. On the other hand, the identification method based on the bat algorithm is introduced to optimize the RF model. Next, we complete the identification task of sitting, standing, raising one’s hands, and falling. Referring to indoor classroom situations, some interference actions are bound to be introduced into each identification task to promote robustness and applicability. The whole optimization scheme is adopted to evaluate the identification effect in the dual Kinect V2 system one by one. The final results show that although some tasks such as the standing identification task are not significantly improved by introducing the method in this paper, other identification tasks are sharply improved. The accuracy can be improved by up to 30.25% at maximum. It can be clearly seen that our method can boost the effect of the HAR task, particularly in flexible orientation scenarios with interference actions.

In the future, our work still has enormous potential. While the Kinect V2 device used in this study has excellent performance, it has limitations in some scenarios indeed. Thus, it may be necessary to pick up more appropriate devices in such situations. With the help of more advanced deep learning theory and the increasing computing ability of mobile terminals, we anticipate that more outstanding models and methods will emerge, providing further inspiration for our future research. While we acknowledge the rigorous spirit and methodical process demonstrated in the production processes of some public datasets, it is important to note that their processes did not consider flexible orientation factors cautiously. To enhance the robustness of our algorithm, we believe it is necessary to refer to their core processes in completing large-scale production of key actions in real-world situations. Furthermore, since the core processes in our method are currently aimed at single-person action identification, if You Only Look Once (YOLO) frameworks or others are introduced in the future, it will be possible to achieve multi-person action detection during each batch in our system. At present, it is worth noting that there remains a considerable disparity in the recognition performance of our method for multi-person actions when compared to the method based on the new deep learning model in both single and multi-person scenarios. Given the introduction of additional peripherals and the reasonable handling of clock synchronization issues, it is worth considering extending the correction mechanism mentioned in this manuscript from single-person to multi-person scenarios. Finally, as for unbalanced multi-classification occasions, we can try to adopt other novel methods to balance or adjust them, which will definitely facilitate the identification methods for human actions. Further consideration will be given to the semantic interpretation of skeleton joint information under that circumstance.

## Figures and Tables

**Table 1:** Comparison of NLOS Localization Signals Identification Methods.

Core Method	NLOS Acoustic Signal Identification Accuracy
Logistic Regression [34,50]	47.19%
SVM (Averaging various kernel functions) [33,50]	61.45%
LDA [50]	55.12%
Decision Tree [34]	69.35%
Random Forest	73.43%
Original AdaBoost (Consists of SVM)	91.50%
*Fuzzy C Method-AdaBoost (Consists of SVM) in this manuscript*	98.13%

**Table 2:** Comparison of Positioning Errors.

Methods	Positioning Accuracy (m)
Average Error in our self-made DoA-Kinect V2 based system	1.50
Method from [33]	0.20
Method from [34]	0.16
*Method in our manuscript*	0.12

**Table 3:** HAR Methods Accuracy Comparison Results.

	Sitting	Standing	Raising One’s Hand	Falling
RF	77.52%	77.54%	79.33%	80.19%
*Bat-RF (part of our methods)*	88.90%	87.93%	80.95%	88.02%
AdaBoost	85.82%	89.79%	82.56%	91.80%
*c-means-AdaBoost (part of our methods)*	87.99%	91.12%	83.11%	93.45%
CNN	86.42%	90.12%	74.79%	85.43%
GCN	92.39%	89.98%	84.69%	94.23%
Temporal shift GCN	95.39%	94.19%	88.23%	96.16%
GCN-CNN	96.56%	97.98%	89.98%	95.15%
Kinetic-GAN	90.15%	94.18%	80.17%	92.15%
BERT-GAN	80.99%	88.23%	82.29%	90.16%
MotionBERT	85.67%	91.25%	83.33%	95.48%

**Table 4:** Four HAR Results in Different Methods.

	Sitting	Standing	Raising One’s Hand	Falling
KNN	61.55%	98.15%	59.65%	81.25%
SVM	73.45%	96.10%	66.70%	79.85%
RF	78.65%	99.25%	73.70%	85.95%
MLP	80.60%	90.40%	80.90%	82.50%
CNN	86.10%	99.45%	87.95%	95.75%
AdaBoost	82.85%	98.95%	83.00%	90.80%
RCN	85.55%	98.20%	82.35%	91.80%
Temporal shift GCN	90.15%	94.72%	84.10%	89.40%
DG-STGCN	92.55%	93.65%	83.60%	90.15%
GCN-CNN	90.05%	99.15%	81.20%	97.25%
BERT-GAN	89.95%	95.95%	82.65%	96.75%
Kinetic-GAN	92.15%	98.95%	89.25%	98.15%
MotionBERT	94.70%	99.20%	88.10%	97.25%
*Our Method*	91.65%	98.75%	89.90%	97.70%

## Data Availability

All of the grants for this manuscript are still in the research phase, and some research data or key codes are currently limited to disclosure within the project team. However, some wav files of the acoustic localization signals and human action skeleton joint data set can be provided without privacy concerns. If necessary, you can contact Rui-Xiang Kan via email (bbklasnic@glut.edu.cn) to obtain the Baidu Netdisk (Baidu Cloud) URL link and then download the files you need. They will be uploaded in a zip file and some useful illustrations can be provided inside. Once the link is inaccessible or disabled, you can contact the authors of this article to obtain the latest link.

## Abbreviations

NLOS	Non-Line of Sight
TCP	Transmission Control Protocol
IoT	Internet of Things
SDKs	Software Development Kits
HAR	Human Action Recognition
IMU	Inertial Measurement Unit
USRP	Universal Software Radio Peripheral
EOD	Exploration Ordnance Disposal
CNN	Convolutional Neural Network
RNN	Recurrent Neural Network
GCN	Graph Convolutional Neural Network
BERT	Bidirectional Encoder Representation from Transformers
GAN	Generic Adversarial Networks
NLP	Natural Language Processing
LOS	Line-of-Sight
SVM	Support Vector Machine
RF	Random Forest
VB	Visual Basic
API	Application Programming Interface
DT	Decision Tree
LFM	Linear Frequency Modulation
OSI	Open System Interconnection
UDP	User Datagram Protocol
MEMS	Micro-Electro-Mechanical System
TDoA	Time Difference of Arrival
GPS	Global Positioning System
LBS	Location Based Services
AWGN	Additive White Gaussian Noise
SVD	Singular Value Decomposition
DoA	Direction of Arrival
YOLO	You Only Look Once

## References

[1] Gao F., Fang W., Sun X., Wu Z., Zhao G., Li G., Li R., Fu L., Zhang Q. (2022). A novel apple fruit detection and counting methodology based on deep learning and trunk tracking in modern orchard. Comput. Electron. Agric..

[2] Liu H., Deng Y., Guo D., Fang B., Sun F., Yang W. (2021). An Interactive Perception Method for Warehouse Automation in Smart Cities. IEEE Trans. Ind. Inform..

[3] Gong L., Wang C. (2019). Research on Moving Target Tracking Based on FDRIG Optical Flow. Symmetry.

[4] Chilo N.O.M., Ccari L.F.C., Supo E., Espinoza E.S., Vidal Y.S., Pari L. (2023). Optimal Signal Processing for Steady Control of a Robotic Arm Suppressing Hand Tremors for EOD Applications. IEEE Access.

[5] Worrallo A.G., Hartley T. (2022). Robust Optical Based Hand Interaction for Virtual Reality. IEEE Trans. Vis. Comput. Graph..

[6] Majumder S., Kehtarnavaz N. (2021). Vision and Inertial Sensing Fusion for Human Action Recognition: A Review. IEEE Sens. J..

[7] Ramirez H., Velastin S.A., Aguayo P., Fabregas E., Farias G. (2022). Human Activity Recognition by Sequences of Skeleton Features. Sensors.

[8] Yu Z., Zahid A., Taha A., Taylor W., Kernec J.L., Heidari H., Imran M.A., Abbasi Q.H. (2023). An Intelligent Implementation of Multi-Sensing Data Fusion with Neuromorphic Computing for Human Activity Recognition. IEEE Internet Things J..

[9] Chen J., Sun Y., Sun S. (2021). Improving Human Activity Recognition Performance by Data Fusion and Feature Engineering. Sensors.

[10] Ramirez H., Velastin S.A., Meza I., Fabregas E., Makris D., Farias G. (2021). Fall Detection and Activity Recognition Using Human Skeleton Features. IEEE Access.

[11] Issa M.E., Helmi A.M., Al-Qaness M.A.A., Dahou A., Abd Elaziz M., Damaševičius R. (2022). Human Activity Recognition Based on Embedded Sensor Data Fusion for the Internet of Healthcare Things. Healthcare.

[12] Cao Y., Xie R., Yan K., Fang S.-H., Wu H.-C. (2022). Novel Dynamic Segmentation for Human-Posture Learning System Using Hidden Logistic Regression. IEEE Signal Process. Lett..

[13] Li M., Wei F., Li Y., Zhang S., Xu G. (2020). Three-Dimensional Pose Estimation of Infants Lying Supine Using Data from a Kinect Sensor With Low Training Cost. IEEE Sens. J..

[14] Bhiri N., Ameur S., Alouani I., Mahjoub M., Khalifa A. (2023). Hand gesture recognition with focus on leap motion: An overview, real world challenges and future directions. Expert Syst. Appl..

[15] Yuwen X., Zhang S., Chen L., Zhang H. (2023). Improved interpolation with sub-pixel relocation method for strong barrel distortion. Signal Process..

[16] Galván-Ruiz J., Travieso-González C.M., Pinan-Roescher A., Alonso-Hernández J.B. (2023). Robust Identification System for Spanish Sign Language Based on Three-Dimensional Frame Information. Sensors.

[17] Wei D., Chen L., Zhao L., Zhou H., Huang B. (2022). A Vision-Based Measure of Environmental Effects on Inferring Human Intention During Human Robot Interaction. IEEE Sens. J..

[18] Tran T., Ruppert T., Eigner G., Abonyi J. (2023). Assessing human worker performance by pattern mining of Kinect sensor skeleton data. J. Manuf. Syst..

[19] Tölgyessy M., Dekan M., Chovanec Ľ. (2021). Skeleton Tracking Accuracy and Precision Evaluation of Kinect V1, Kinect V2, and the Azure Kinect. Appl. Sci..

[20] Mansoor M., Amin R., Mustafa Z., Sengan S., Aldabbas H., Alharbi M.T. (2022). A machine learning approach for non-invasive fall detection using Kinect. Multimed. Tools Appl..

[21] Kuriakose B., Shrestha R., Sandnes F. (2023). DeepNAVI: A deep learning based smartphone navigation assistant for people with visual impairments. Expert Syst. Appl..

[22] Moon S., Park Y., Ko D., Suh L. (2016). Multiple Kinect Sensor Fusion for Human Skeleton Tracking Using Kalman Filtering. Int. J. Adv. Robot. Syst..

[23] Chhetri S., Alsadoon A., Al-Dala’in T., Prasad P., Rashid T.A., Maag A. (2021). Deep learning for vision-based fall detection system: Enhanced optical dynamic flow. Comput. Intell..

[24] Apicella A., Snidaro L. (2021). Deep Neural Networks for Real-Time Remote Fall Detection. Proceedings of the International Conference on Pattern Recognition, Virtual, 10–15 January 2021.

[25] Cheng K., Zhang Y., He X., Chen W., Cheng J., Lu H. Skeleton-Based Action Recognition with Shift Graph Convolutional Network. Proceedings of the 2020 IEEE/CVF Conference on Computer Vision and Pattern Recognition (CVPR).

[26] Duan H., Wang J., Chen K., Lin D. PYSKL: Towards Good Practices for Skeleton Action Recognition. Proceedings of the 30th ACM International Conference on Multimedia.

[27] Duan H., Wang J., Chen K., Lin D. (2022). DG-STGCN: Dynamic Spatial-Temporal Modeling for Skeleton-based Action Recognition. arXiv.

[28] Ramirez H., Velastin S.A., Cuellar S., Fabregas E., Farias G. (2023). BERT for Activity Recognition Using Sequences of Skeleton Features and Data Augmentation with GAN. Sensors.

[29] Degardin B., Neves J., Lopes V., Brito J., Yaghoubi E., Proenca H. Generative Adversarial Graph Convolutional Networks for Human Action Synthesis. Proceedings of the 2022 IEEE/CVF Winter Conference on Applications of Computer Vision (WACV).

[30] Xu L., Song Z., Wang D., Su J., Fang Z., Ding C., Gan W., Yan Y., Jin X., Yang X. (2022). ActFormer: A GAN Transformer Framework towards General Action-Conditioned 3D Human Motion Generation. arXiv.

[31] Shahroudy A., Liu J., Ng T., Wang G. (2016). NTU RGB+D: A Large Scale Dataset for 3D Human Activity Analysis. arXiv.

[32] Liu J., Shahroudy A., Perez M., Wang G., Duan L., Kot A. (2020). NTU RGB+D 120: A Large-Scale Benchmark for 3D Human Activity Understanding. IEEE Trans. Pattern Anal. Mach. Intell..

[33] Kan R., Wang M., Zhou Z., Zhang P., Qiu H. (2022). Acoustic Signal NLOS Identification Method Based on Swarm Intelligence Optimization SVM for Indoor Acoustic Localization. Wirel. Commun. Mob. Comput..

[34] Kan R., Wang M., Liu X., Liu X., Qiu H. (2023). An Advanced Artificial Fish School Algorithm to Update Decision Tree for NLOS Acoustic Localization Signal Identification with the Dual-Receiving Method. Appl. Sci..

[35] Seifallahi M., Mehraban A., Galvin J., Ghoraani B. (2022). Alzheimer’s Disease Detection Using Comprehensive Analysis of Timed Up and Go Test via Kinect V.2 Camera and Machine Learning. IEEE Trans. Neural Syst. Rehabil. Eng..

[36] Li B., Zhang C., Han C., Bai B. (2019). Gesture Recognition Based on Kinect V2 and Leap Motion Data Fusion. Int. J. Pattern Recognit. Artif. Intell..

[37] Kwolek B., Kepski M. (2014). Human fall detection on embedded platform using depth maps and wireless accelerometer. Comput. Methods Prog. Biomed.

[38] Tran T., Le T., Pham D., Hoang V., Khong V., Tran Q., Nguyen T., Pham C. A Multi-Modal Multi-View Dataset for Human Fall Analysis and Preliminary Investigation on Modality. Proceedings of the 2018 24th International Conference on Pattern Recognition (ICPR).

[39] Adhikari K., Bouchachia H., Nait-Charif H. Activity Recognition for Indoor Fall Detection Using Convolutional Neural Network. Proceedings of the 2017 Fifteenth IAPR International Conference on Machine Vision Applications (MVA).

[40] Liu C., Hu Y., Li Y., Song S., Liu J. (2017). PKU-MMD: A large scale benchmark for continuous multi-modal human action understanding. arXiv.

[41] Martínez-Villaseñor L., Ponce H., Brieva J., Moya-Albor E., Núñez-Martínez J., Peñafort-Asturiano C. (2019). UP-Fall Detection Dataset: A Multimodal Approach. Sensors.

[42] Hansen L., Salamon P. (1990). Neural network ensembles. IEEE Trans. Pattern Recognit. Mach. Intell..

[43] Breiman L. (1996). Bagging predictors. Mach. Learn..

[44] Breiman L. (2001). Random forests. Mach. Learn..

[45] Salim H., Alaziz M., Abdalla T. (2021). Human Activity Recognition Using the Human Skeleton Provided by Kinect. Iraqi J. Electr. Electron. Eng..

[46] Abobakr A., Hossny M., Nahavandi S. (2018). A Skeleton-Free Fall Detection System from Depth Images Using Random Decision Forest. IEEE Syst. J..

[47] Freund Y., Schapire R. (1996). Experiments with a New Boosting Algorithm. Machine Learning, Proceedings of the Thirteenth International Conference, San Francisco, CA, USA, 3–6 July 1996.

[48] Huang X., Li Z., Jin Y., Zhang W. (2022). Fair-AdaBoost: Extending AdaBoost method to achieve fair classification. Expert Syst. Appl..

[49] Avidan S. Spatialboost: Adding Spatial Reasoning to AdaBoost. Proceedings of the 9th European Conference on Computer Vision.

[50] Zhang L., Huang D., Wang X., Schindelhauer C., Wang Z. (2017). Acoustic NLOS Identification Using Acoustic Channel Characteristics for Smartphone Indoor Localization. Sensors.

[51] Hazra S., Pratap A., Nandy A. (2023). A Novel Probabilistic Network Model for Estimating Cognitive-Gait ConnectionUsing Multimodal Interface. IEEE Trans. Cogn. Dev. Syst..

[52] Wang Y., Wu Y., Jung S., Hoermann S., Yao S., Lindeman R. (2021). Enlarging the Usable Hand Tracking Area by Using Multiple Leap Motion Controllers in VR. IEEE Sens. J..

[53] Wang Y., Chang F., Wu Y., Hu Z., Li L., Li P., Lang P., Yao S. (2022). Multi-Kinects fusion for full-body tracking in virtual reality-aided assembly simulation. Int. J. Distrib. Sens. Netw..

[54] Yang X., Gandomi A. (2012). Bat algorithm: A novel approach for global engineering optimization. Eng. Comput..

